# A Mixed-Methods Examination of Fertility Desires and Reproductive Planning Among Low-Income Black Fathers

**DOI:** 10.1007/s40615-025-02663-8

**Published:** 2025-10-16

**Authors:** Adaobi Anakwe, Wilson Majee, Abigail Olajire, Rhonda BeLue

**Affiliations:** 1 Dornsife School of Public Health, Drexel University, Philadelphia, PA USA; 2 Department of Health Sciences, University of Missouri, Columbia, MO United States; 3 Department of Communication, University of Missouri, Columbia, MO United States; 4 College for Health, Community and Policy, University of Texas San Antonio, San Antonio, TX United States

**Keywords:** Reproductive life planning, Black fathers, Sexual and reproductive health, Fertility desires, Mixed methods

## Abstract

**Background:**

Reproductive life planning (RLP) supports the achievement of fertility goals for both men and women, yet most studies focus on women. Black fathers living with low income, a vulnerable population at risk for multiple unplanned pregnancies and negatively stereotyped as reproductively irresponsible, are rarely asked about their RLP. This study aimed to examine the relationship between Black fathers’ fertility desires and pregnancy prevention strategies and explore their contextual nuance.

**Methods:**

A concurrent mixed-methods approach was conducted using quantitative data (*n* = 36) and qualitative data (*n* = 17) among Black fathers living with low income and participating in a Midwestern urban community–based fatherhood program. Differences between groups were compared using Fisher’s exact test, and thematic analyses examined the relationship between drivers of participants’ fertility desires and reproductive planning practices.

**Results:**

Participants had a median age of 33 years. Findings indicated that participants’ fertility desires were not associated with pregnancy prevention (*p* = 0.251). Most *interview participants desired pregnancies* and recognized the *need for a reproductive life plan.* Health before pregnancy and financial stability were important *determinants of fertility planning*, and using *pregnancy contraception* was dependent, among other factors, on navigating trust with a partner. Overall, participants thought that *using an adult mindset*, in which they considered the consequences of unprotected sex, was key to family planning.

**Conclusion:**

Findings highlight a breadth of factors influencing Black fathers’ RLP and underscore the need for robust, tailored services. Community-based organizations already working with Black fathers can be leveraged to increase reproductive health awareness and facilitate reproductive life planning.

## Background

Reproductive life planning (RLP), defined as a set of personal goals regarding the decision on whether (or not) to have children, supports the achievement of fertility goals for both men and women [1, 2]. An alignment of RLP components (including the desire for children and corresponding contraception use) should lead to either a planned pregnancy or active pregnancy prevention. However, while extant literature continues to focus on promoting RLP for women [3], men’s fertility desires (i.e., whether (or not) they want to have children) are largely neglected. Yet, in some contexts, men’s fertility desires are the greater determinants of fertility achievement among couples [4, 5]. These studies suggest that whereas women who do not want to have a pregnancy are more likely to utilize contraception consistently and effectively, men’s fertility desires and contraception use are misaligned.

Men’s fertility desires, whether for a(nother) child *or* considering their partners’ pregnancy as unintended, mistimed, or unwanted [6], are associated with paternal involvement before, during, and after pregnancy (mistimed pregnancies were associated with lower levels of paternal visitation), child outcomes and well-being (unintended pregnancies were associated with poorer child mental proficiency), parent–child relationship (unintended pregnancies were associated with increased mother-father conflict), and parenting style and health outcomes for men themselves (unintended pregnancies were associated with increased depressed symptomology among fathers) [7–10]. Further, unintended pregnancies among men were associated with poorer health outcomes and risk-tolerant social behaviors, particularly within “at-risk” populations such as the economically disadvantaged, ethnic, or racial minorities [11, 12]. Men’s fertility desires are also a stronger determinant of rapid repeat pregnancies (i.e., pregnancies occurring within 24 months apart), over and beyond the mother’s pregnancy desires [13], which has negative health consequences for mothers and children. Conversely, men who desire a pregnancy are also more likely to maintain better health behaviors preconceptionally, provide in-cash or in-kind support to their pregnant partner(s), and report overall better health for themselves and child(ren) [8, 14]. Thus, while it is known that men’s fertility desires impact their own reproductive wellness and their partner’s and child’s health, not enough is known about how these desires translate to men’s RLP and even less about this relationship among Black adult fathers. Per the Centers for Disease Control and Prevention (CDC), understanding men’s fertility desires is key to ensuring that all pregnancies are planned and wanted [15].

### Black Men and Reproductive Life Planning

Where Black men are socially located at the intersection of “Black” and “being a father” and “living with low income” may influence their fertility desires and actions toward RLP. Despite their greater involvement with their children [16], tropes of Black fathers, as deadbeat and reproductively irresponsible, are pervasive, especially for those living with low incomes [17]. These societal attitudes continue to shape Black men’s fathering experiences. In the USA, Black men disproportionately live on low income, experience earlier onset of sexual activity, and have a longer fertility window than their White counterparts (i.e., 3% of Black men experience their first births by age 15–19 years compared to 1.2% Whites of the same age) [11]. Further, they disproportionately bear children with multiple sexual partners, are less likely to live with their biological child(ren), and are also less likely to receive formal contraceptive education [11, 18]. Black fathers living with low income bear steeper consequences of unplanned pregnancy, including penalties from defaulting on child support, compared to their White counterparts [19]. Although these inequities and fathering contexts are rooted in the historical disenfranchisement of this population [20, 21] and continue to shape the context in which Black men (and fathers) make reproductive decisions, they are rarely considered in RLP research. Structural determinants, including lifetime lower socioeconomic mobility and poorer access to preventive services, consequently increase Black men’s risk for unintended pregnancies, sexually transmitted infections (STIs), and underutilization of RLP [1, 22–26]. Policy environments also impact Black men’s RLP. For instance, the 2022 Dobbs decision that rescinded the constitutionally protected right to abortion [27] created an additional barrier to accessing reproductive health care, especially for households living with low income—forcing families to continue unintended pregnancies with greater exposure to health risks, especially in states that enacted abortion bans [28, 29].

Summarily, Black men’s use of and support for women’s contraception is shaped by a combination of multilevel factors, including differences in fertility desires between partners, attitudes toward contraception, inter-partner communication about fertility goals, cultural and gender norms, availability of male-focused family planning services, and lack of attention to men in reproductive health policies [30]. The tenets of reproductive justice, i.e., the recognition of the human right to bodily autonomy to decide when and whether to have children, necessarily require that we grapple with historically unjust practices at the intersections of gender, race, and class that have perpetuated reproductive harm in Black communities [31, 32]. Reproductive injustices in the USA have contributed to invisibility and insensitivity to Black men’s reproductive health needs. For example, the exploitation of Black men in reproductive health research, exclusion from beneficial interventions [33, 34], and the role of public health and health care providers in shaping Black men’s orientation toward reproductive wellness remain understudied historical factors that likely impact Black men’s current participation in RLP.

Despite the demonstrated importance of RLP among Black fathers living with low income due, in part, to their vulnerability to multiple unintended pregnancies [35], they are rarely asked to provide insight on their RLP. The aims of this study were, therefore, to examine the relationship between Black fathers’ fertility desires and pregnancy prevention strategies and to explore the contextual nuances of their reproductive life planning.

## Methods

### Study Design and Study Setting

A concurrent mixed-methods design was used in this study to quantitatively examine the relationship between Black fathers’ fertility desires and pregnancy prevention strategies and qualitatively explore the nuances of this relationship. Both data strands were collected simultaneously and integrated during analysis and interpretation to provide a comprehensive understanding of Black fathers’ RLP [36]. Previous literature highlighted complexities with aligning fertility desires/intentions and actual behavior; thus, both approaches were necessary to elicit deep insights. Survey and interview questions were adapted from the reproductive life planning (RLP) protocol and tailored to the study population. The RLP protocol comprises a set of questions recommended by the CDC that are used to identify an individual’s reproductive goals and support intentional reproductive decision making [1, 37–39]. This protocol can be used in clinic and community settings [40]. Questions from this protocol were adapted for both survey and interview processes [36].

### Sampling and Recruitment

Following ethical approval from the lead author’s institution, 36 Black fathers were recruited from a community-based fatherhood program in a Midwestern urban city serving families living with low income. This community was selected for its large and diverse Black population, high prevalence of unintended pregnancies [41], and proximity to the authors. The community organization provides comprehensive programs and services, including family formation, legal aid, employment mentoring, youth development, and peer support. Eligible participants were biological fathers aged 18 or older, enrolled in the program, and consented to participate. Typically, the program receives a new cohort of fathers each month. Recruitment occurred monthly from February to July 2021, with study presentations made during each family formation program session. Presentations were made via Zoom, and participants were recruited from five cohorts. To achieve our research objectives, we utilized a convenience sample recruited through the oldest and largest responsible fatherhood organization in the city, serving predominantly Black fathers living with low income. A $30 gift card was offered as an incentive.

#### Quantitative Survey

The quantitative component involved a 23-item online survey assessing demographic characteristics, fertility desires, and pregnancy prevention behaviors. Fertility desire was measured by asking participants if they wanted children in the future, with response options “yes,” “no,” or “mixed feelings.” Pregnancy prevention behaviors were assessed via two questions. One question measured whether participants were trying to prevent a pregnancy, with response options “yes,” “no,” and “maybe,” and the other question assessed the method of pregnancy prevention used on a Likert scale ranging from no prevention to vasectomy.

#### Qualitative Interviews

For qualitative data, 17 participants agreed to participate in individual interviews or focus groups conducted via Zoom or in person, adhering to pandemic guidelines. The first author scheduled a mutually agreeable time with each participant and received verbal consent. Of the seventeen participants, eleven chose to participate in individual (1:1) interviews, and six participated in two focus group (four fathers in one group) interviews. Every effort was made to address the challenges in engaging Black fathers by providing different modalities from which fathers could select. Each father in the focus group was given an opportunity to answer interview questions independently. Interviews lasted 45–90 min and followed a semi-structured guide developed in collaboration with the community partner (see Table 1).

**Table 1:** Interview protocol for study participants

Domains	Questions	Probes
Domain 1: Introduction and fatherhood perceptions		
1	Can you tell me a little about yourself? Where did you grow up? How long have you lived in this community? What does family and community life mean to you?	What made you join the program
2	When you think about “fatherhood,” what comes to mind?	
3	When you think about family, having children (or the children you had), what are your thoughts about the number of children to have as a family?	
4	What was/were your biggest concerns about raising children?	
Domain 2: About your health as a man and father		
5	When you think about “being healthy,” what comes to mind	
6	When you think about your health, what are some challenges you face with access to health care?	If/when you go to the doctor, what questions would you typically ask?
		What other resources do you access to meet your health needs?
7	Thinking in terms of your health, how would you say you are doing?	If a doctor or health care provider ever told you (or were to tell you) to take care of yourself, how did (would) you feel?
8	Given these thoughts around your health, how do you think it affects your child?	
9	Thinking in terms of your health or the health of men in general, what would you like to change before having children?	What efforts would you make toward changing this/these behavior/s?
		What recommendations can you make to change things that are beyond your control?
Domain 3: Male contraceptive use		
10	What are your thoughts on contraception? Feel free to discuss with examples from your experience	What are some challenges you face with accessing and using contraception?
11	Can you tell me about how much discussion you have with your partner around family planning? (Contraceptive use? Condom use? Number of children?)	–
12	How has your experience as a father been impacted by COVID-19?	
13	Is there anything else about your health or your desire to have children you want to talk to me about?	

Because of the sensitivity of reproductive health topics, albeit of minimal risk to our study participants, we followed data management best practices that included storing deidentified data on the lead authors’ institutions’ HIPAA-compliant, physically and digitally encrypted devices.

### Analysis

#### Quantitative

Survey data were analyzed using simple univariate and bivariate descriptive statistics. Frequency percentages were computed; differences between men’s fertility desires and pregnancy planning behaviors were compared using Fisher’s exact tests. This test is suitable for comparing proportional differences in categorical data with a small sample size [42]. Pregnancy planning behaviors were assessed in two ways: (a) whether (or not) a father was actively preventing pregnancy and (b) what method was used for prevention (none, partner uses at least one method, I always use condoms, I had a vasectomy, or I do not know). We hypothesized that men’s fertility desires would be associated with pregnancy prevention. Significance testing to detect statistically significant differences between groups was set at *p* < 0.05. A complete case analysis was used because missingness accounted for less than 3% of the data. All analyses were completed using SPSS.

#### Qualitative

All interviews were transcribed verbatim and thematically analyzed. Three researchers independently reviewed the first four transcripts and drafted a codebook inductively. The researchers met to discuss any areas of disagreement until a consensus was reached; the codebook was finalized. Two researchers then coded the remaining interviews deductively, applying the codes from the codebook. A final meeting examined all coded transcripts, verified completeness, ensured reliable coding, and resolved areas of disagreement [43]. Transcripts and codes were entered into Dedoose for further analysis [44].

## Results

### Descriptive Statistics

Thirty-six participants completed the survey; the median age was 33 years (range: 23 to 63 years). About 90% had more than a high school education, 38.9% had full-time employment, and 50% were never married or in a marriage-like relationship. Most men (58.3%) had only one child, and the majority (72%) did not reside in the same home with their child(ren). Men’s desire for a future pregnancy was almost evenly split between positive, negative, and ambivalent desires (yes = 38.9%; no = 30.6% and ambivalent = 30.6%). However, most men said they were actively preventing pregnancy (69.4%), with the most prevalent contraceptive method being condoms (52.8%) (Table 2).

**Table 2:** Demographic characteristics of study participants (*n* = 36)

Socio-demographic characteristics	Frequency	Reproductive health and planning characteristics	Frequency
	*n* (%)		*n* (%)
Age (median)	33 years	Future fertility desires	
Marital status		No	11 (30.6%)
Never married	18 (50)	Yes	14 (38.9%)
Divorce	2 (5.6)	Ambivalent	11 (30.6%)
Separated	4 (11.1)	Actively preventing pregnancy	
Cohabiting	8 (22.2)	No	8 (22.2)
Married	4 (11.1)	Yes	25 (69.4)
Educational attainment		Maybe	3 (8.3)
< HS	4 (11.1)	Pregnancy prevention method	
HS	11 (30.6)	None, planning to have a baby	2 (5.6)
Some college	13 (36.1)	My partner uses	5 (13.9)
2-year degree	8 (22.2)	I always use a condom	19 (52.8)
Employment status		Had a vasectomy	1 (2.8)
Not employed	12 (33.3)	Don’t know	8 (22.2)
Part time	8 (22.2)	Number of sexual partners	
Full time	14 (38.9)	None	11 (30.6)
Other	2 (5.6)	One	19 (52.8)
Income level		Two	2 (5.6)
No income	5 (13.9)	Three or more	4 (11.1)
$1–$9999	15 (41.7)	Most comfortable discussing my sexual and reproductive health	
$10,000–$24,999	5 (13.9)	Parent	7 (19.4)
$25,000–$49,999	11 (30.6)	Sibling	2 (5.6)
Number of biological children		Partner	9 (25.0)
1	21 (58.3)	Health provider	15 (41.7)
2	5 (13.9)	Other	2 (5.6)
3	4 (11.1)		
4	3 (8.3)		
5 or more	3 (8.3)		
Residential parent			
Yes	9 (25.0)		
No	26 (72.2)		
General health			
Poor	1 (2.8)		
Fair	7 (19.4)		
Good	11 (30.6)		
Very good	11 (30.6)		
Excellent	6 (16.7)		
Health insurance			
Yes	15 (41.7)		
No	21 (58.3)		
Length of participation in the program			
< 6 weeks	29 (80.6)		
6 weeks	1 (2.8)		
> 6 weeks	6 (16.7)		

*n* < 36 due to missing information

Demographic characteristics are based on ***all*** participants who consented to participate

### Bivariate Analysis

Table 3 shows the results from the bivariate analysis examining the relationship between Black fathers’ fertility desires and pregnancy prevention using Fisher’s exact test. We found no statistically significant association between fertility desires and active pregnancy prevention (*p* = 0.251); nor was a statistically significant association found between fertility desires and the method used to prevent pregnancy (*p* = 0.174).

**Table 3:** Bivariate analysis examining the relationship between men’s fertility desires and pregnancy prevention

	Fertility intentions			Fisher’s exact test
	No	Yes	Ambivalent	*p*-value
	*n* (%)	*n* (%)	*n *(%)	
Actively preventing pregnancy				0.251
No	2 (18.2)	4 (28.6)	2 (18.2)	
Yes	9 (81.8)	7 (50.0)	9 (81.8)	
Maybe	0 (0.0)	3 (21.4)	0 (0.0)	
Prevention method				0.174
None, planning to have a baby	0 (0)	2 (14.3)	0 (0)	
My partner uses	1 (10)	0 (0.0)	4 (36.4)	
I always use a condom	5 (50.0)	9 (64.3)	5 (45.5)	
Had a vasectomy	1 (10)	0 (0)	0 (0)	
Don’t know	3 (30.0)	3 (21.4)	2 (18.2)	

### Qualitative

Of the 36 participants, 17 completed qualitative interviews—six in two focus groups and 11 as individual interviews. The acronyms IN and FC are used to denote the format of participants’ interviews (IN = individual, one-on-one; FC = focus group). Emergent themes were classified into five domains. These themes explored participant perspectives on their fertility desires and pregnancy planning behaviors and explained how different factors influence the relationship between them. Emerging themes suggested that Black fathers may (or may not) (1) *desire future pregnancies* and (2) *recognize the need to have a reproductive life plan*. However, (3) *additional contextual factors influence their fertility desires*, and (4) *even more factors influence actions taken toward pregnancy prevention*. Overlying these themes, participants acknowledged the need to (5) *utilize an adult mindset* to align their fertility desires with their pregnancy prevention behaviors.

### Emerging Themes

#### Theme 1: Black Fathers May (or May Not) Desire Future Pregnancies

Black fathers’ desire for pregnancy seemed to occur on a continuum, ranging from those who did not want additional pregnancies to those who were unsure of their future fertility desires. Most men, however, desired a future pregnancy as one participant noted, “I do want more children…” (IN821). Among participants who desired additional pregnancies, it seemed that most wanted to experience multiple births—that is, have more than one child (see narratives from IN721 and IN621). Some Black men desire “as many children as I can handle” (IN621) irrespective of their partner’s affirmation or other potentially limiting factors; “there is no number for them” even as many as “10 kids” (IN721).

Conversely, some participants were ambivalent toward a subsequent pregnancy. One participant noted, “We [my partner and I] did have a conversation about that…after this child. I’m not really sure how I feel about having any more kids” (IN282)*.* However, the strongest factor deterring the desire was the impact of age on remaining actively involved with their offspring and/or providing for them. All referred sample quotes are provided in Table 4.

**Table 4:** Detailed narratives and qualitative themes from Black fathers living with low income and participating in a fatherhood-focused community–based program

Themes	Subthemes	Sample quotes
Theme 1 Black fathers may (or may not) desire future pregnancies	–	I want to have about 10 kids—-. For real, [my female partner(s)] don’t get too excited about that. They really don’t get too excited about that. They would like maybe one or two, but my limit is too much for them. (IN721)
		For me–for me, no children are planned for me. I’m too old for that. [Laughter]… Now if a child was coming now at his age from when they graduate, nah, uh-uh. I still want to be active. I would like to have been active and stuff like that in a child’s life so, you know, in eight years I’ll be 60, you know, so no, not for me. FC121
Theme 2 Black fathers recognize the need to have a reproductive life plan	–	For me, what would I have liked to have changed, my first child was not planned. But fortunately, I was in a financial position to be able to handle that. So, I would have liked for it to been able to be more part of that plan, [i.e., have a plan at the time]. (IN111)
		Man, gettin’ to know who they are as a person, goals, plans. You must have goals. I didn’t have no goals before I had children. I didn’t have no plan before I had children. I was lackin’ of communication. Goals, plans, and communication are everything when you’re talkin’ about children. You cannot mention children and not have no goals. You cannot mention children and not have no plans. You cannot mention children and not have no communication. And you cannot mention children and not be willin’ go build and bond with the person that you lookin’ to have a child with. (IN921)
		the environment I grew up in, my cousin, he had a lot of women. He’s got like 12 children now. You know, me not being educated a little earlier from older men and also women. Like I didn’t start doing anything [having sex] ‘til I was 17 and when my grandma found out, she like… here, you’re getting this [condoms]. I’m like, oh, Lord, okay. And then she told the doctor one day. The doctor say here are some condoms. I said okay. I think it’s more so, me not having the right people in front of me, you know, growing up around a woman, I guess, I was trying to find a woman. (IN182)
		Yeah, the thing with family planning, [you need to] put out what’s your values and like what your partner’s values and see if they match it. You know, if not, try to come to a middle ground in a way that we both agree with. (IN821) We had a talk as soon as after having him [their son], that this would be our only one. And if we had any down the road, that would be a different talk. (FC262)
		We talk about how we don’t, well, both of us don’t believe in abortion. We don’t believe in that. (IN282)
Theme 3 Black fathers’ fertility desires and reproductive planning behaviors are influenced by several factors	Neighborhood characteristics	The environment here is not really conducive [for raising a family] – well, depending on where you at. I grew up in the hood and stuff like that. Personally, I don’t want to raise a family or bring up a family in the places that I grew up. And I feel like the only way for me to be successful in that is relocating. It don’t even have to be out of state. I mean, it can be literally in another part. I want to make sure that the environment is conducive… I want to be in the right community and the right environment that’s gonna play an important part, you know, in having a healthy family. (IN221)
	Financial stability and readiness	I would like my financial status to change first and foremost [before having children], ‘cause in order to have more children you’ve got to have the bread and bacon, you know, to feed all them mouths… (IN221)
		First of all, time and affordability, are the two elements that go into planning for children because it’s the greatest sacrifice… that you really have to make when you talk about being a parent. So, I’m looking at, we have to look at when is the best time, when am I willing to and ready to make the time commitment of that it’s going to take to parent these children. And with that… being able to financially support them; home, transportation, food, clothing, other activities. What type of lifestyle do we want to have, vacationing, those types of extracurricular activities for the children so it comes into really putting a plan together based on, and part of your life plan of when you want to make the commitment… (IN111)
	Characteristics of the current partner(s)	No, if I ever [wanted] another child, it’d be another woman…I know I ain’t gonna have another child by my first kids’ mother, and if I gotta deal with the second situation like as I did with my first kid, oh no, I’m cool on kids… if I find me a good woman in my life to where we bonded and buildin’, I would not wanna give a woman a child…But just right now I’m dealin’ with the women I’m used to dealin’ with, no, I’m cool on kids. But like I said, if I meet me somethin’ different that I never had in my life, that shows me differently, I would love to wanna have another child bein’ the fact that my two kids would be older and they know what type of daddy they got to the point where now they can kinda sorta like be a big sister, big brother to daddy’s third child. But other than that, until I find that good woman, I’m just – I’ll be thankful for the two kids I got. (IN921)
	Preconception/inter-conception health	Mental health, being more healthier, and increasing just overall health, as a whole, I want that to be definitely one thing… I would love that to have been changed [before becoming a father]. (IN221)
		I feel like, before I even have a kid, I need to be in a good mental state or that’s going [to], affect the children. So you know, I got to correct myself first before, you know, I can correct anybody else …Just make sure you’re the best version of yourself that you can before you have children, I mean. And that’s mentally, physically. I mean everything accounts for that. You know, financially. emotionally. Everything. Just make sure you’re the best version of yourself before you have a child so that won’t affect the children nor the partner you’re having the child with. (IN821)
		I suffered from a drug addiction for a while. I was addicted to crack and heroin. But through a lot of changes, especially with my people, places, and things, my environments, I was able to seek help and find it. I wish that I didn’t smoke, and that I exercised more. (IN021)
Theme 4 Several factors influence Black fathers’ actions toward pregnancy prevention	Knowledge of contraception use	I took a lot of chances and thanks be to God. In the heat of the moment, I really didn’t know how to use them [condoms]. Going to get some grease anyway, and [using grease] just make the condom bust. I learned that from being in this program. (FC121)
		I thought about a vasectomy. I thought about it…, I don’t want to do it…I still need to be able to use [my penis], would I have trouble going to the restroom? Like, how would that work? Like, what would be the effects of it? Would I become incontinent or dysfunctional? I can’t have none of that. (IN312)
		We need more resources, programs, where they just talk about sex ed. We don’t have enough sex ed program, and I think a lot of people would probably go sit in the class if they knew all this stuff [STIs] that’s going around there…. We’re misguided as a people. We could definitely use programs out there that get focused on sex ed. Just like how you be in high school and have health class, you know? Where they talk about sexual education. (IN202)
	Men’s attitudes and behaviors toward pregnancy prevention	When I’m talking to family members and friends, I’m 100% for contraceptive use. I’m the best giver of advice but I don’t follow my own advice when it comes to that area, because I may can start with one [condom], then to me it [does not feel] right. So, it’ll be like, hey, can I take this off. And they’d [a partner] be like yeah, okay. You know, stuff like that, and then I don’t end up using it. …then the female condom, that’s so unattractive. The way it looks! I might as well take a Family Dollar bag and go to the bathroom. (FC121)
		I said I got over my lust and my love. So, it’s like it’s no reason for me to do anything with anybody that I’m not going to be with for a long time or doesn’t want to be here for a long time [or]… at least be something to my kids…. I don’t believe in vasectomy or abortion. (IN621)
		I more so would expect for the woman to kind of take care of that with the depo shots, you know, Norplant and things like that. That’s an up and down battle [male condom use], because I might can start with it, don’t mean I’m going to finish it. Or, if I don’t bring enough and another round starts… (FC121)
		You know, setting that standard upfront of like contraceptives or any other like troubling matter and, you know, holding that expectation throughout the relationship. …if your partner feel they need that with you [contraceptive], that standard should hold throughout the relationship until, we come to another meeting and… potentially changing that expectation. (IN821)
	Navigating trust with a partner	Well, if you really don’t trust your partner and if they not really tell you the whole story of their sexual history before you guys get into that sort of thing, or if you even feel that your partner is not faithful and, going out besides you guys, and having sex with other people out there. So that’s what I mean as, trustworthy. (IN821)
		I feel that if you’re in a committed relationship, say for, I don’t know, a year and a half, two years, three years, something like that, and you and your partner are comfortable and trust each other enough, I feel like you don’t have to use protection if you don’t want to, but then you run the risk of pregnancy. (IN282)
		You really have a blind trust. You know, she said she clean; I say I’m clean; okay. But we really don’t know. And, in all essence, at the end of the day we really truly don’t know …But then when it comes to the foreplay, everything is all good… So, what is the contraceptive for? So that’s just strictly to prevent pregnancy because it’s not- to prevent STDs or STIs, because if it was then, we would use it even on the oral side. So, a lot of time to me that comes a conflict like don’t have me go down raw and then say, ‘Put it on.’ What? Well, you already had something; now I’ve already got it. So, if I ain’t using it in that fashion [before vaginal sex], then my conflict is I’m not going to use it in this fashion either [during vaginal sex]. (FC121)
		We just talk about, hey, have you checked for any STDs? Do you have a clean slate? What are your thoughts on condoms? What are your thoughts – you know, getting tested together. I try to make my partner feel comfortable with it as well. I wouldn’t try to single my partner out and be like, "Oh, you’re the one to get all this stuff; I’m not gonna do nothing [get tested]." No, let’s do this together so – we both in the same boat. I try to definitely make my partner comfortable, when it comes to like the testing and safe sex and all that. (FC121)
		And so, when we get to talking about a relationship, and sexual practices that’s a business part of a relationship type of conversation, no more than if we’re going on a date [and asking], okay, who’s paying? That’s a business conversation in a relationship. So, the same thing should go for sex. In most cases [for this participant], there’s conversation going on about sex before you go lay down. (IN111)
Theme 5 Have an adult mindset		People don’t care until it happens. There’s no preventative measures, ain’t no contraception until somebody’s pregnant. (IN202)
		First, start with the female that you choose to engage with. You should want someone that has some type of future aspirations and a plan. Instead of just doing something with someone willy nilly. Don’t put your future in anyone else’s hands, use the contraceptive, it’s your life, you know, so you don’t hand over control of your life or take the risk about your life, by not protecting yourself….. " And then I say maturity. Are you able to comprehend and understand that more mature mindset about the impact. We’re talking about a life, and the impact that this could have on your life…So [understanding] the full impact of the responsibility, that’s built into that goes with sex, not just the pleasure and enjoyment…What is it you want to accomplish in life? Where are you trying to go and what’s it gonna take for you to get there? Does a child fit into that plan… with the mindset of the sacrifice that will be necessary for you to make and commit to a child in terms of time, and finance. (IN111)

#### Theme 2: Black Fathers Need a Reproductive Life Plan

The CDC recommends that men have a reproductive life plan in order to achieve their fertility desires and maximize reproductive health and outcomes [15]. Most men in this study deemed it necessary to have a reproductive plan; however, some men noted they learned the value of planning a pregnancy after they became fathers (IN111, IN921). The perception of reproductive planning is often intentionality, communication with a partner, and a willingness to “build and bond with the person that youlookin’to have a child with,” (IN621) because anything else would be an unintended pregnancy. Most importantly, in retrospect, they thought that having (a) proper education about the value of reproductive planning, (b) adequate positive role-modeling (especially from significant male figures), and (c) the right support networks to foster uptake of pregnancy prevention methods were necessary to prevent unplanned pregnancies (IN182, IN821). Specifically, this participant believed that if he and his cousin (a father of 12) were given timely, accurate sexual education, “having the right people in front of me” (IN182), perhaps he would have made better reproductive health decisions.

Overall, participants thought that congruence between a couple’s fertility desires was central to reproductive planning. They emphasized that congruence should be achieved through shared reproductive goals and decision making, including discussions about fertility desires (FC262, IN282). They recognized the need for reproductive planning through alignment of partners’ values and communication to arrive at a “middle ground.”

However, while participants were aware of the benefits of RLP, our quantitative findings revealed a mismatch between their desires and pregnancy prevention strategies, suggesting that certain factors hinder them from translating their knowledge into action.

#### Theme 3: Several Factors Influence Fertility Desires and RLP

Although most participants deemed it necessary to have a reproductive plan, they identified several factors influencing their fertility desires and their agency in planning a pregnancy. For these participants, important factors included their place of residence, current financial situation, their partner (at the time), and their health status before a(nother) pregnancy. As all participants were already fathers, they reflected on these factors retrospectively and underscored the value of considering their fertility desires within these contextual factors.

### Neighborhood Characteristics

Participants noted that one’s neighborhood should be an important consideration when planning to start a family. One participant noted that living in high-crime, high-poverty, and low-socioeconomic areas that lacked a sense of community or resources (such as schools) to cater to their families’ needs would stymie their desire for more children. When thinking of having a(nother) child, “…we [he and his partner] have conversations about the school district, where we want to raise them [our child/future children].” (IN282). Another participant desired to have more children, but through comparing his past life in “the hood” to his current living situation, he affirmed that residing in an “environment [that] is conducive” and in the “right community” is an important predictor of his reproductive planning—which he does not have now (IN221).

### Financial Stability and Readiness

In addition to neighborhood contexts, participants felt that being financially prepared was central to developing an RLP. They voiced that the number of children a man desires to have “kind of dependson your financial situation,” which should align with “how you’re able to provide for those… children.” (IN821). In addition to pregnancy timing, participants believed that their ability to afford basic needs, as well as other extracurricular leisure activities, such as vacation, required some level of commitment from both partners.

### Characteristics of the Current Partner(s)

The person a man is currently partnered with was also an important determinant of fertility desires and reproductive planning. One participant noted that he needed to have a partner different than his child’s mother (current partner)—a partner more aligned with his values would increase his likelihood of having a future pregnancy (IN921).

### Preconception/Inter-conception Health

Participants stated that good preconception health was a necessary ingredient for reproductive planning. Reflecting on what they could have done differently before transitioning to fatherhood and what they want to do before becoming fathers again, men emphasized the need to be healthier. Specifically, participants spoke to the need and value of optimal mental health (IN221, IN821). Both participants defined good health before pregnancy as being mentally stable and “healthier” for the child, because only a mentally stable individual can properly raise or correct a misbehaving child. They also considered it necessary to prioritize their well-being and have a plan for maintaining good health. One said, “Make sure you got a plan for your health…don’t stop taking care of yourself, you know, because you gotta take care of somebody else*.*” (IN182). They also emphasized the need for a healthy diet (IN821).

Many fathers lamented some of the choices that they had made earlier that compromised their health because, as one participant noted, “I feel like I wasn’t loving myself enough when I had my first two, when I had my kids in marriage. I’d say I wasn’t loving myself enough. So, like being that you don’t love yourself you really cannot like love anybody” (IN621). Since becoming fathers (and in considering future fatherhood), these participants were intentionally taking actions to modify their health behaviors. One participant noted that changing his social structure and environment helped him improve his behavioral and physical health (IN021), and he believes those are requisites for having more children.

#### Theme 4: Several Factors Influence Pregnancy Prevention

RLP should reflect an alignment between fertility desires and the use/non-use of pregnancy prevention strategies. Along these lines, participants highlighted factors influencing their pregnancy prevention, which included their (1) knowledge of contraception, (2) attitudes toward contraception and condom use, and (3) navigating trust with a partner.

### Knowledge of Contraception Use

Despite clearly understanding the need for an RLP, participants felt that their inconsistent/ineffective use of contraception was attributable to inadequate condom and contraception knowledge in the Black community. One participant noted, “I feel we don’t use them enough…we need a lot of education about condoms and contraception because the Black community do not really use them. We really don’t.” (IN202). Some participants felt they lacked specific knowledge on correct condom use. For instance, participants spoke of using oil-based lubricants with latex condoms or foregoing condoms completely (FC121). The family formation program clarified best practices with participants.

Further, among fathers who were either ambivalent toward pregnancy or did not desire additional pregnancies, there seemed to be a lack of knowledge and misconceptions about vasectomies and pregnancy prevention. These men seemed fearful and uncertain of the procedure and its effects (IN312). Other participants highlighted the need for programs in schools and within the Black community to educate youth and community residents about consistent and effective contraception—including information about STIs (IN202).

### Men’s Attitudes and Behaviors Toward Pregnancy Prevention

Black fathers’ attitudes toward pregnancy prevention varied widely. Some felt that some form of contraception should be used all the time if they did not desire additional pregnancies, “…you should take all the preventative measures they have available if you really don’t want to have kids with somebody” (FC121), but others struggled with consistent use. In the moment, these men prioritized their sexual enjoyment over pregnancy prevention, particularly when with a consenting partner. One participant noted that contraception is important to the extent that he advises his family members and friends to utilize them, though he is not willing to sacrifice his pleasure in favor of condoms, even if the consequence is having an unintended pregnancy (FC121).

Among other participants, there seemed to be an ambivalence toward pregnancy prevention. As one father noted, “…in my head, me saying, okay, I don’t care, … if I have a kid, I’m having a kid.” (IN182). However, several participants thought abstinence was the best policy and were trying to stay celibate “…I’m not having sex, and if I do it, it’s going to be protected and that’s just all” (IN202). For some of these men, abstinence was rooted in religious beliefs and personal values, which seemed to influence their perspectives on using contraception. One participant’s beliefs about abortion and vasectomy influenced his decisions to sacrifice temporal pleasure with a short-term partner (IN621).

Whereas many participants considered their intrinsic needs and values as driving their attitudes toward contraception, some fathers felt it was up to their female partner(s) to prevent pregnancy. Male condoms were perceived as more inconvenient—“I feel like men don’t really like condoms because how tight they are. You lose feeling…I didn’t wanna wear them, so it wasn’t nothing really stopping me from using ‘em, but it was me stopping me…” (IN202). Another participant believed that it is his partner’s responsibility to use non-condom contraception because he can choose to remove a condom. In essence, if the woman gets pregnant, it is solely her fault (FC121).

Conversely, some participants’ attitudes toward pregnancy prevention were rooted in partnership and communication, believing that prior to sexual intercourse, partners should establish shared values and goals in line with any consequences of unprotected sex, such as an unintended pregnancy. One participant stated that the contraception plan established at the beginning of a relationship must be followed until both partners agree to change expectations (IN821).

### Navigating Trust with a Partner

Participants felt that partner trust influenced their attitudes toward contraceptive use and pregnancy prevention. Some suggested that condom use signified a lack of trust in their partner (and relationship) and that practical steps to demonstrate a partner’s trustworthiness were needed, such as confirmatory STI testing: “I don’t really believe in condoms because then you don’t trust the person that you’re with or you haven’t got them tested” (IN621). Participants defined trust as partners’ openness to discuss past sexual relationships and faithfulness to a monogamous relationship (IN821). For some participants, trust was assessed based on how long they were in a relationship with their partner. The longer they were in that relationship, the more likely they were to trust their partner and, in turn, less likely to use condoms (IN282). Although trust seemed to play a significant role in how and whether men practiced safe sex, ultimately, participants acknowledged that this trust was often blind (FC121).

Research has highlighted the need to examine the discussions men have with their partners prior to sexual intercourse. For fathers in this study, their choices to discuss protection before or during a sexual encounter with their partner(s) were influenced by their perceived trust in that partner. In other words, although these men understood the consequences of unprotected sex, the way it translated into their dealings with a potential partner still rested on trust, usually through a verbal confirmation that their partner did not have an STI (FC121).

Some men highlighted their need for consistent messaging from their partner regarding condom use during sex. However, the bigger concern for men in these conversations seemed to be the potential to contract an STI and not the need to prevent pregnancy per se. Further, they seemed to indicate that sexual and reproductive health (SRH) was not their responsibility but their partner(s) (FC121).

For other men, condom use was a topic discussed in partnership, prioritizing the feelings of their partner and sharing responsibility for their SRH (FC121). Still, others felt that to prioritize their SRH, the discussion should resemble a business transaction devoid of sentiment and emotion (IN111).

#### Theme 5: Have an Adult Mindset

Most men in this study acknowledged the consequences of unprotected sex, especially the increased likelihood of contracting an STI, but it seemed they only began to consider their SRH/RLP after the fact, i.e., after they contracted an STI or experienced an unintended pregnancy—“people don’t care” or take precautions until a pregnancy occurs or an STI is contracted (IN202).

Overall, participants spoke of the need to have an adult mindset, taking responsibility for RLP early: “And the adult mindset would have been to use condoms, which could have prevented me from having a child at 21[years]. So, had I just **used my adult mindset** back then, [I] could have prevented a whole lot of this now” (FC262). Specifically, men felt they needed to take greater ownership of their reproductive health beginning with (a) who they engaged as a sexual partner, (b) examining their reproductive goals relative to their material conditions and future plans, and (c) aligning their fertility desires and pregnancy prevention (or planning) with these goals (IN111).

## Discussion

This pilot study applied a reproductive life planning (RLP) lens to explore fertility desires and pregnancy prevention among Black fathers living with low income in a Midwestern US urban area. Black fathers’ fertility desires were unrelated to their pregnancy prevention efforts. Qualitatively, we found that Black fathers’ fertility desires were influenced by perceptions of their neighborhood context, financial stability, pre- or inter-conception health, and their current partners’ characteristics. Men’s actions toward pregnancy prevention were also influenced by their knowledge about and attitudes toward utilizing pregnancy prevention methods. Predominantly, men felt that the extent to which they trusted their partner played a key role in whether they used contraception and how they had conversations about safe sex. Ultimately, men in this study thought that to align their fertility desires with their pregnancy prevention behaviors, they needed to utilize an adult mindset in which they considered the consequences of unprotected sex prior to engaging in any sexual activity.

Although about a third of men in this study did not desire another pregnancy, their fertility desires were not associated with their utilization or method of contraception. These findings are consistent with previous studies that highlighted incongruence between fertility desires among men living with low income and contraception use [23]. Our findings contrast with RLP perspectives, which suggest that fertility desires should align with pregnancy prevention efforts to ensure that all pregnancies are wanted and planned, mitigating the cascading long-term effects of unintended, mistimed, and unwanted pregnancies [2]. Our findings reemphasize the need for RLP efforts tailored to this population. Also, since Black fathers may have the dual role of preventing unintended pregnancies for themselves and socializing their sons on SRH [45], they remain a critical group to engage in public health efforts to mitigate unintended pregnancies.

In our study, the majority of Black fathers desired multiple births, notwithstanding the deterrent of age, underscoring the centrality of pregnancy to Black men and corroborating previous literature on biological age as a determinant of fertility desires [46–48]. Previous research suggests that men are happier with achieving a pregnancy (independent of intention status) than women [10], which likely contributes to rapid repeat pregnancies [13].

We identified significant contextual determinants of Black fathers’ fertility desires. A man’s habitation, financial stability, characteristics of his partner, and health status before a pregnancy played key roles in the desire for additional pregnancies. These factors are consistent with conceptualizations of extrinsic factors that can influence men’s fertility desires [47, 49]. Previous studies have highlighted the primacy of financial readiness in men’s fertility desires [23]. In contrast to adolescent males who favored unintended pregnancies if they lived in low-income neighborhoods [49], our findings highlight that older Black fathers prioritized leaving poorer neighborhoods to start a family.

The extant literature agrees with our findings that inadequate contraception knowledge, discomfort with using condoms, and misconceptions about vasectomies are fundamental barriers to pregnancy prevention among Black men. For instance, Campbell and colleagues demonstrated that men living with low income aged 24–55 years had limited contraceptive knowledge and perceived their risk of causing a pregnancy to be low [46]. For some of our participants, religious beliefs and personal values were key drivers of pregnancy prevention. Aversion to medical interventions like abortion and views of condomless sex as natural continue to hinder pregnancy prevention efforts among men [22]. Further, some Black men associated condom use with a lack of trust in their partner, which is consistent with previous literature [50].

Consistent with studies among women [51], we found that men’s desire for another pregnancy was linked to their current partner; if a partner’s characteristics aligned with their values and they were open to condomless sex, men were more inclined to trust that partner and less likely to prevent pregnancy. Although relationship characteristics such as trust in a partner, length of the relationship, and commitment strength were previously identified as important contributors to consistent condom use [52], little is known about specific partner characteristics most pertinent to childbearing or the extent to which perceptions of conceiving a child with the “wrong” partner contribute to desiring a pregnancy. Sexual trust contributes significantly to whether and how sexual dyads engage in condomless sex, yet often symbolic talk such as “are you clean?” is used rather than risk-informed cognitive assessments [50]. This is why studies have highlighted the need for open discussion among dyads on sexual health [53]. Although men in our study engaged in these conversations, they seemed to be driven by perceptions of partner trust, aligning with previous literature [5].

Lastly, our study participants felt that in order to align their fertility desires with pregnancy prevention, they needed an adult mindset in which they considered the consequences of unprotected sex beforehand. These findings are consistent with previous studies among Black men who highlighted the need to use their “big head [brain] over your little head [penis],” in order to protect themselves from HIV and other STIs [54]. However, men were more reflective about RLP after they became fathers. They recognized the disproportionate burdens associated with having children in the context of their structural realities and the need for intentional RLP. As noted by Jackson and colleagues, Black men are reproductively responsible, but the realities of their social positioning (e.g., lower socioeconomic status, reproductive wellness, education, health access, incarceration) pose significant barriers to family planning in this population [55].

### Policy, Practice, and Research Implications

Stereotypes of Black fathers often portray them as deadbeat and reproductively irresponsible, yet little has been done to understand how they navigate RLP. These men lack material resources and agency to adequately plan for pregnancy, increasing their risk for additional unintended pregnancies. Our findings challenge negative tropes of Black fatherhood: These men clearly understood the need for RLP but are navigating other contextual factors that may be stronger determinants of their fertility desires and pregnancy prevention. Our study, therefore, prompts the question: In what ways can we ensure that Black men are supported to plan pregnancies in culturally sensitive, contextually relevant ways that mitigate structural constraints to their fuller participation in RLP?

Most men in our study only realized the importance of RLP after becoming fathers, suggesting that more efforts are needed to engage men earlier in SRH and fertility planning. Reproductive health interventionists and family planning practitioners should intentionally inquire about Black fathers’ fertility desires and contextual factors influencing their behaviors from a perspective that hones their desire to be responsible fathers. Doing so requires a recognition of historical racialized patriarchy that has induced societal dismissal of Black men’s fertility and parenting interests.

Educational interventions that provide sexuality education to Black men in culturally grounded ways are needed. For instance, many fatherhood-focused community organizations already engage at-risk fathers in financial education but seldom integrate SRH. These organizations should be leveraged as community partners in reproductive health, instead of relying on clinic settings. Given Black men’s well-founded historical mistrust of the medical system [56, 57], an approach that meets them where they are already engaged will be useful.

It is important to create policies that are sensitive to men’s fertility desires as well as women’s. Gender-sensitive polices accounting for the reproductive health needs of all should be developed. In the wake of state policies restricting contraceptive use and access, especially in predominantly Black communities living with low income, it will be critical to research the impact on Black men’s uptake of these services.

Finally, understanding Black fathers’ motivations to engage in pregnancy prevention can highlight key areas for developing culturally sensitive intervention points. Multi-partner fertility is common among Black fathers and is often negatively stereotyped. Men in our study prioritized their partner’s characteristics over the pervasive heteronormative value of marital childbearing that is upheld as the “gold standard” for “proper” childbearing and rearing [58, 59]. Reproductive health policies and interventions that condemn multi-partnered fertility as irresponsible and inherent to Black fathers while ignoring their motivations will fail to generate any sustainable positive impact. The community-based framework recently developed by Hall and colleagues that integrates contraception and preconception care is beneficial for Black fathers [3].

### Strengths and Limitations

This study applied a mixed-methods approach to explore a seldom-examined phenomenon in a vulnerable population, providing needed insight about factors contributing to incongruence between fertility desires and pregnancy prevention among Black fathers living with low income. However, our study has some limitations. We focused exclusively on a population of Black fathers living with low income enrolled in a fatherhood-focused program in the US urban Midwest. It may not be generalizable to other men living with low income in other geographical areas and not in a fatherhood-focused program. Participants were already engaged in a comprehensive fatherhood-focused program that may have helped them re-evaluate the importance of reproductive planning. We selected a wide age range of men (anyone older than 18 years), which may contribute to the heterogeneous perspectives in our findings. Although all participants were fathers, younger men may have significantly different views, experiences, and expectations from older fathers. We did not account for the age or number of children participants had, which may be important when considering their desires for a future pregnancy. Additionally, we had a small sample size for our quantitative analysis, which lacked adequate power to perform any advanced statistical analysis.

## Conclusion

Black fathers living with low income may (or may not) desire additional pregnancies. Although they recognize the need for RLP, their fertility desires were not related to their pregnancy prevention efforts. Financial readiness, neighborhood characteristics, pre- or inter-conception health, and the perceived characteristics of a partner were important contextual determinants of fertility planning. Actions taken toward pregnancy prevention were influenced by men’s knowledge about and attitude toward contraception. However, perceived trust in a partner was central to men’s decisions to use contraception, even though this trust was often blind. Men thought that they needed to use an “adult mind set” before engaging in sex to consider the consequences and align their fertility desires and pregnancy prevention efforts. Future public health and policy efforts should tap into Black fathers’ values and address the contextual factors influencing their reproductive planning to mitigate the burden of unintended pregnancies. Community-based organizations already working with Black fathers can be leveraged to increase reproductive health awareness and facilitate reproductive planning for fathers.

## Data Availability

The data used in this study is available upon reasonable request from the first author.
